# Assessing progress in the national health financing system towards universal health coverage in Iran: a mixed-method study protocol

**DOI:** 10.1186/s12961-020-00610-z

**Published:** 2021-01-12

**Authors:** Mina Anjomshoa, Ali Akbari Sari, Amirhossein Takian

**Affiliations:** 1 Department of Health Management and Economics, School of Public Health, Tehran University of Medical Sciences, Tehran, Iran; 2 Department of Global Health and Public Policy, School of Public Health, Tehran University of Medical Sciences, Tehran, Iran; 3 Health Equity Research Centre (HERC), Tehran University of Medical Sciences, Tehran, Iran

**Keywords:** Health financing system, Universal health coverage, Iran

## Abstract

**Introduction:**

Health financing systems have a key role in achieving universal health coverage (UHC) across the globe. However, little is known about how best to monitor health financing system progress towards UHC, especially in low- and middle-income countries. This is a protocol of a study that will aim to assess health financing system progress towards achieving UHC in Iran.

**Methods:**

An explanatory mixed-method approach will be used in two phases. In the quantitative phase, the performance of the Iranian health financing system will be assessed using a well-established set of indicators to draw on progress over 5-year intervals starting in the year 2000 up to the present. Data will be extracted from the global health expenditure database using a specific form and will be classified in accordance with each indicator. A qualitative phase will then take place considering the Kutzin et al. framework and by using health financing progress matrices. The qualitative phase will consist of two successive stages; first, a descriptive overview on the major health coverage schemes along with key attributes of each scheme. This initial mapping will be the underlying background for the second stage. In the second stage, the matrices comprised of a series of questions and relevant to the core functions of health financing and cross-cutting options will be invested in enhancing the evaluation of the ongoing reforms or policies. In this phase, data will be collected by reviewing national policy documents and in-depth interviews with key informants who will be recruited using purposive sampling. Finally, a policy discussion with key stakeholders will be held in order to review and verify the consistency between the current health financing policy and UHC goals.

**Discussion:**

This study will provide a comprehensive image about the current status of the national health financing system progress towards achieving UHC in Iran. Such assessment will give detailed insight about the performance of the current financing system through identifying encountered challenges. Furthermore, some other defects in the design of the financing system are expected to appear. In all likelihood, the results will be fruitful enough to make informed decisions about interventions and policies in relation to UHC.

**Ethics and dissemination:**

The study protocol has been approved by the Ethics Committee for Research at Tehran University of Medical Sciences. Informed consent will be obtained from all key informants and the data will be collected and transcribed anonymously in order to maintain utmost confidentiality. The results will be disseminated in peer-reviewed journals and presented in national and international conferences and meetings.

## Introduction

In September 2015, the United Nations General Assembly introduced a broad and universal policy agenda entitled Transforming Our World: The 2030 Agenda for Sustainable Development. This agenda has embraced 17 sustainable development goals (SDGs) involving 169 integrated and comprehensive targets. SDG3, encompassing 13 interrelated targets, has been adopted based on its endeavour to ensure healthy lives and promote well-being for all people at all ages [1, 2]. Stemmed from the Alma Ata declaration, universal health coverage (UHC) became one of the intrinsic targets generally, and particularly in SDG3 [3–5], as it contributes to improving the access of individuals and communities to essential health services [6, 7].

**Fig. 1:**
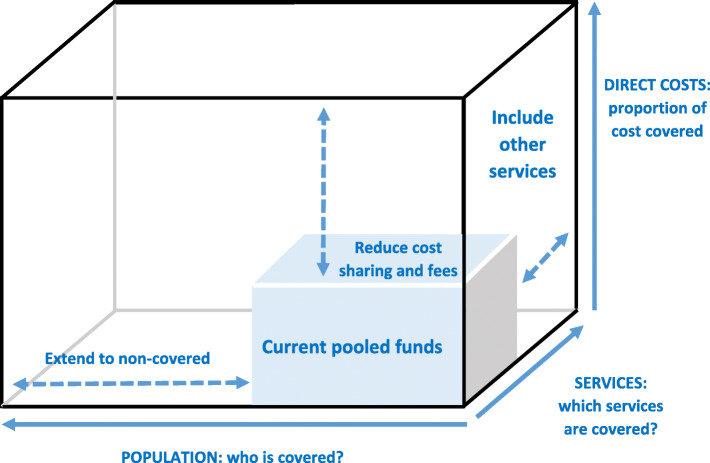
Universal health coverage (UHC) cube

According to WHO, UHC has been accurately defined as a desired health system outcome that reflects its capacity in responding to all people’s needs, any time and anywhere, without financial hardship. UHC includes a full range of services such as promotion, prevention, treatment, rehabilitation and palliative care [8]. It should be noted that UHC does not necessarily mean the provision healthcare services for free, rather, it focuses on enhancing access without financial hardship [9]. Accordingly, the ‘UHC cube’ was used in the World Health Report 2010, and reflects three distinct dimensions of coverage, namely population coverage, service coverage and financial coverage [10] (Fig. 1). These three dimensions of the cube represent the policy choices that might be prioritised and implemented to address the gap in the answers of the three critical questions of who is covered, which services are covered and how much is paid out-of-pocket [11]. Therefore, UHC as a concept, can act a key role in accomplishing three main goals of the health system, that is, equity in access to health services, quality of care and financial protection [11]. Health system strengthening is one of main pillars when moving towards UHC, and needs countries to work on this aim [7, 12]. In order to do so, special attention should be paid to the building blocks of the health system, including governance, service delivery, financing, workforce, medicines and technologies, and information [2, 12, 13]. Undoubtedly, health financing is of a particular importance through influencing the three UHC dimensions of equity, quality, and financial protection as well as of the SDG3 overall [12]. Hence, United Nations member countries have highlighted health financing as a principal factor to avoid financial hardship and reach the universal coverage [6].

Little is known about the best way to assess the capacity of health financing systems to achieve UHC. To achieve this, we have to grasp the current situation and know what is needed to bring about UHC into reality [14]. Systemically, the assessment of health financing systems is an invaluable guideline for policy dialogue underpinning the national health plan related to UHC and its budget in particular [5].

**Fig. 2:**
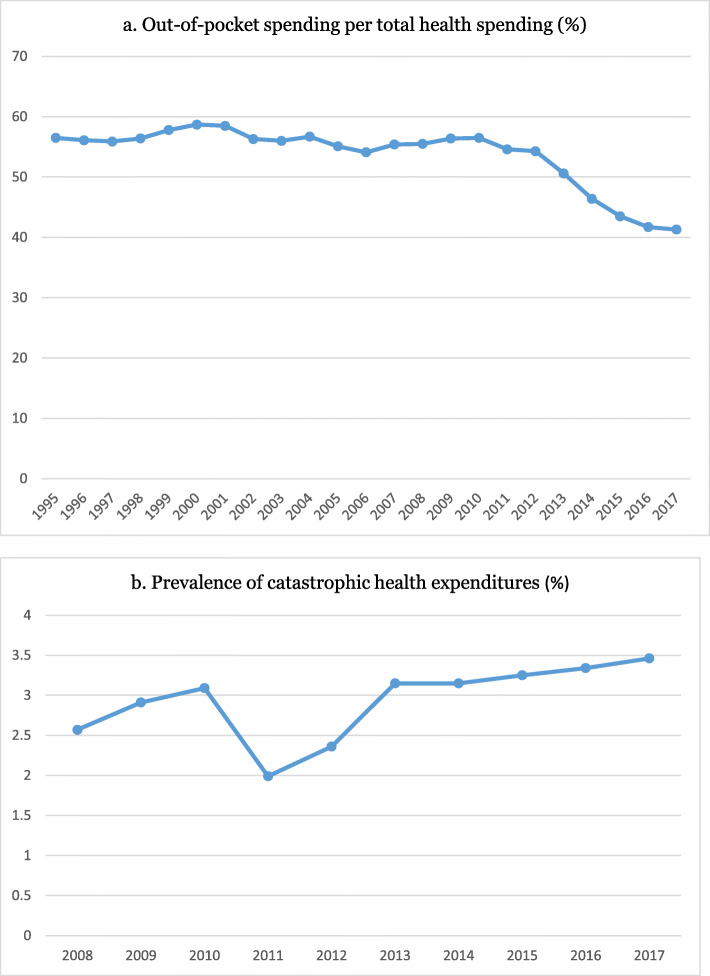
Prevalence of out-of-pocket spending and catastrophic health expenditures in Iran

**Fig. 3:**
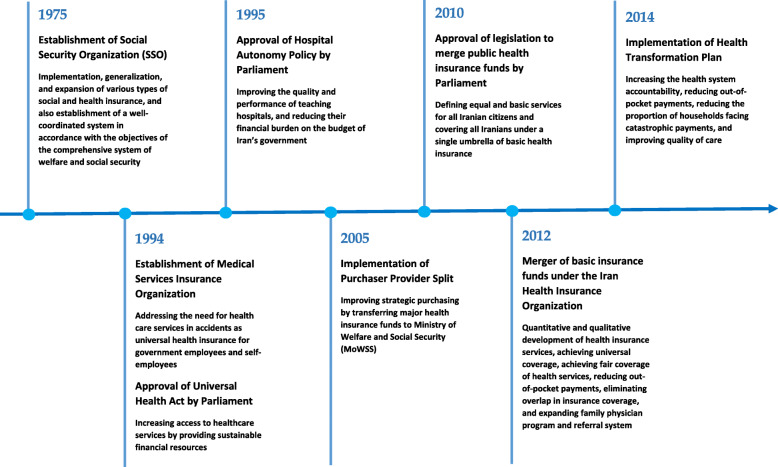
Key health financing system reforms and their objectives in Iran

According to the joint report released by WHO and the World Bank in 2017, Iran is categorized as a low-performing, low- and middle-income country with respect to financial protection [2]. Figure 2 describes the financial protection indicators in Iran and their trends during the last years. Although, UHC was prioritized in the country to be achieved by 2025 [15], Iran has encountered many challenges and financial hardship [15–30] (Table 1). To cope with these circumstances, the government has launched a wide range of health financing reforms in the recent years (Fig. 3).

**Table 1:** The challenges of health financing system in Iran

Health financing challenges	References
Catastrophic costs	[16–19]
Low level of sustainability	[15, 20–22]
Deficit and expensive payment system, relied on fee-for-service	[15, 23–25]
Inadequate share of the public sector in total health expenditures	[16]
Fragmentation of revenue sources and pooling	[15, 23, 24, 26]
Low level of efficiency	[24]
Existence of informal payments	[27, 28]
Increased treatment costs	[16]
Ineffective strategic purchasing mechanisms	[15, 29, 30]
Insufficient redistribution of cross-subsidy between the insurance schemes	[15]
Differences between tariffs of public and private services	[16]
Poor transparency of financial flow	[23, 24]

To the best of our knowledge, this is the first study to assess the capacity of the national health financing system for achieving UHC in Iran. The results of this study might  assist policy-makers to design new policies towards achieving UHC in Iran.

## Methods

### Study design

**Fig. 4:**
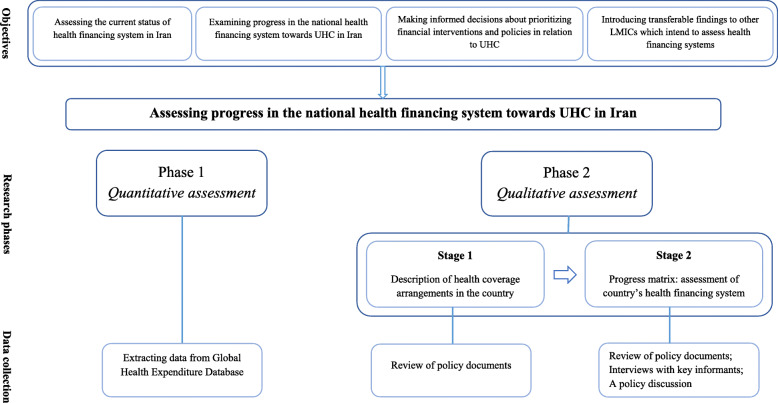
Breakdown of research phases

This explanatory mixed-method study will be comprised of two consecutive phases, namely a quantitative and a qualitative phase. The study design, phases, data collection, and analysis are illustrated in Fig. 4.

### Quantitative phase

Based on our systematic search, we will draw on a set of health financing indicators, introduced by WHO in December 2019 (Additional file 1) [31]. This set of indicators has been adopted from the global health expenditure database (GHED), which depends on the framework of System of Health Accounts 2011 [32, 33]. These indicators are helpful in estimating financial flows, especially financing sources and schemes. In addition, they will propose the rationale, definition and suitable measurement methods to monitor the progress towards UHC, evaluate the impact of health reforms, and compare the results with those of other countries [32].

Data will be extracted from GHED using a specific form designed for this purpose; these data will then be classified according to each indicator. The annual growth rate according to health financing indicators will be presented in 5-year intervals starting from the year 2000. This study will descriptively analyse the secondary aggregated health financing data about Iran. All data will be analysed using STATA. We will generate scatter plots and graphs, and simple calculations will be used to determine the percent change in health financing trends.

### Qualitative phase

Theoretically informed by the Kutzin et al. framework [34], qualitative assessment will be conducted using Health Financing Progress Matrices (HFPMs) [35], which have been developed to appraise the consistency between health financing policy developments and UHC objectives [35, 36]. These matrices were introduced by the Department of Health Systems Governance and Financing of WHO in December 2019 in order to formulate a framework valid for monitoring and assessing health financing systems. As a result, assessment findings will be combined with information derived from the quantitative phase in order to get a comprehensive and detailed depiction of the current health financing system aims at achieving UHC in Iran.

**Fig. 5:**
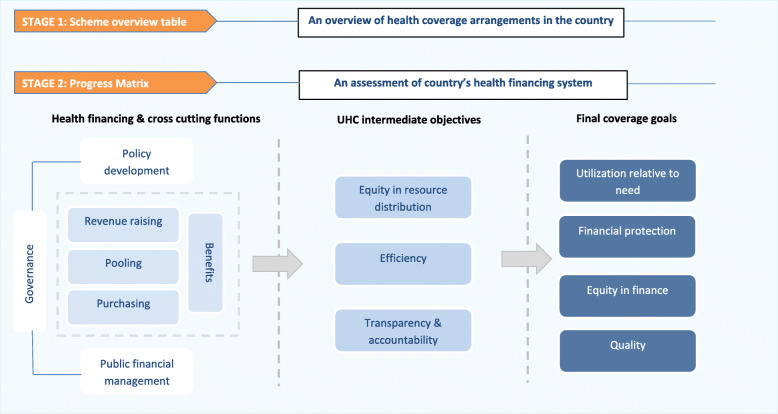
Stages and components of health financing progress matrix

Qualitative assessment will be undertaken through two successive stages. In the first stage, a descriptive overview of the major health coverage schemes in the country will be outlined along with key attributes of each scheme; thus, this initial mapping will stand as a basis for the second stage. In the second stage, the matrices comprised of a series of questions and relevant to the core functions of health financing and cross-cutting options will be invested in enhancing the evaluation of the ongoing reforms or policies. Figure 5 summarizes the stages and components of HFPMs [37].

The required information will be mostly obtained from secondary sources with careful referral to the supporting documents or related interviews. Reviewing the national documents will be useful in updating knowledge about the health financing policy regarding its formulation, underlying evidence and key actors, and identification of areas in need of revision.

In-depth, face-to-face, and semi-structured interviews will be conducted with key informants in the country, e.g. health financing experts, policy-makers and academics, using an interview guide published by HFPMs. Key informants will be recruited using a purposive sampling technique, and the time and place of interview will be assigned by direct coordination with interviewees. We estimate that approximately 15 interviews will be needed; subsequently, the recruitment of new key informants will continue up to saturation. It is estimated that each interview will last 60–90 minutes.

Informed consent will be obtained from the participants and the interviews will be recorded using a digital recorder and transcribed verbatim directly after the interview. Policy documents and transcripts will be thematically analysed using MAXQDA, a software developed to facilitate data management in qualitative research. Finally, a policy discussion with key stakeholders will be held in order to review and verify the consistency between the current health financing policy and UHC goals.

### Ethics

The study protocol has been approved by the Ethics Committee for Research at Tehran University of Medical Sciences. Informed consent will be also obtained from key informants; data will be collected anonymously to maintain utmost confidentiality.

## Discussion

Although there is a global commitment to the UHC approach [38], it has not been fully achieved in any country around the world. Nevertheless, most countries have made relative progress and moved towards UHC-relevant goals [11]. Most countries are deeply working on strengthening their systems to progress towards UHC [7, 12]. Health system strengthening includes enhancing all components of the health system that are central to moving towards UHC, including governance, service delivery, financing, workforce, medicines and technologies, and information [2, 12]. In particular, the component of health financing has proved to be essential in ensuring effective progress towards UHC, as it impacts three intermediate UHC objectives, namely, efficiency, equity and transparency, and thereby ultimately contributes to the achievement of UHC elements cited in SDG target 3.8 [12]. It is noteworthy that robust health financing structures are essential for achieving UHC [5, 39]. To improve the formulation and implementation of health financing policies, it is fundamental to assess the current status to identify strengths and weaknesses in addition to opportunities and threats. Such assessment will provide a clear profile about the performance of the existing system and the demanded reforms before navigating towards UHC.

This study is going to fill in a remarkable gap in the literature through usage of a mixed-methods design examining the ongoing status as well as the progress in the national health financing system towards achieving UHC in Iran. To the best of our knowledge, this is the first study that uses two structured frameworks [31, 37] to assess the Iranian health financing system. By using GHED for monitoring health financing indicators, a quantitative assessment of the health financing system will be undertaken, and trends and changes needed will be acquired. The HFPM framework, as a guide for qualitative assessment, will assist in collecting, organising and interpreting the data in a structured and systematic way.

Although there were several previous attempts to reform the national health financing system, some other defects in its design are expected to appear within the findings of this study. IN all likelihood, the results will be fruitful enough to make informed decisions about financial interventions and policies in relation to UHC in Iran. This will also provide an opportunity to assess whether the existing health financing system is performing well or poorly, a diagnosis of the reasons why, and the challenges the country faces in moving towards UHC and will provide the ‘starting point’ for a national health financing reform strategy.

Moreover, this study is anticipated to introduce transferable findings to other low- and middle-income countries that will embark on assessment and reforming of their health financing systems in order to achieve UHC. The results will be disseminated in peer-reviewed journals and presented in appropriate forums, including national and international conferences and meetings. A series of policy briefs will also be introduced to interested national policy-makers in order to promote the successful design and implementation of national health financing policies.

## Supplementary information

**Additional file 1.** Health financing indicators

## Data Availability

Not applicable.

## Abbreviations

UHC: Universal health coverage
GHED: Global Health Expenditure Database
HFPMs: Health financing progress matrices
SDGs: Sustainable Development Goals
