# Supplementary material for: Assessing progress in the national health financing system towards universal health coverage in Iran: a mixed-method study protocol
Source: Health Res Policy Syst. 2021 Jan 12;19:4. doi: 10.1186/s12961-020-00610-z (PMC7805138; doi:10.1186/s12961-020-00610-z)
Supplement: Supplementary file 1 — Additional file 1. Health financing indicators [file 12961_2020_610_MOESM1_ESM.pdf]

Additional file 1. Health Financing Indicators

Indicator Name	Abbreviation	Numerator and Denominator
AGGREGATES		
Current Health Expenditure (CHE) as % Gross Domestic Product (GDP)	CHE%GDP	(HF.1 + HF.2 + HF.3 + HF.4 + HF.nec) / GDP
Current Health Expenditure (CHE) Per Capita in US$	CHE_PC_USD	CHE / Population / XRT
Current Health Expenditure (CHE) Per Capita in PPP Int$	CHE_PC_PPP	CHE / Population / PPP
Capital Health Expenditure (HK) as % Gross Domestic Product (GDP)*	HK%GDP	HK / GDP
Capital Health Expenditure (Domestic Public) as % Gross Domestic Product (GDP)*	HK_GGHED%GDP	HK_GGHED / GDP
Capital Health Expenditure (External) as % Gross Domestic Product (GDP)*	HK_EXT%GDP	HK_EXT / GDP
Total Health Expenditure (THE) as % of Gross Domestic Product (GDP)	THE%GDP	(HF+HK) / GDP
Expenditure by FINANCING SOURCES		
General Government Health Expenditure (GGHE) as % General Government Expenditure (GGE)	GGHE%GGE	(FS.1 + FS.2 + FS.3) / GGE
General Government Health Expenditure (GGHE) as % Gross Domestic Product (GDP)	GGHE%GDP	(FS.1 + FS.2 + FS.3) / GDP
Domestic Health Expenditure (DOM) as % of Current Health Expenditure (CHE)	DOM%CHE	(FS.1 + FS.3 + FS.4 + FS.5 + FS.6 + FS.nec) / CHE
Domestic General Government Health Expenditure (GGHE-D) as % Current Health Expenditure (CHE)	GGHED%CHE	(FS.1 + FS.3) / CHE
Domestic General Government Health Expenditure (GGHE-D) as % General Government Expenditure (GGE)	GGHED%GGE	(FS.1 + FS.3) / GGE
Domestic General Government Health Expenditure (GGHE-D) as % Gross Domestic Product (GDP)	GGHED%GDP	(FS.1 + FS.3) / GDP
Domestic General Government Health Expenditure (GGHE-D) per Capita in US$	GGHED_PC_USD	(FS.1 + FS.3) / Population / XRT
Domestic General Government Health Expenditure (GGHE-D) per Capita in PPP Int$	GGHED_PC_PPP	(FS.1 + FS.3) / Population / PPP
Government Budget Transfers to Social Health Insurance (SHI-G) as % of Social Health Insurance (SHI)	SHI_G%SHI	(FS.1.2 + FS.1.1xHF.1.2.1) / HF.1.2.1 – ***create FS.1.1xHF.1.2.1***
Self-Employed Contributions to Social Health Insurance (SHI-SE) as % of Social Health Insurance (SHI)	SHI_SE%SHI	FS.3.3 / HF.1.2.1
Domestic Private Health Expenditure (PVT-D) as % Current Health Expenditure (CHE)	PVTD%CHE	(FS.4 + FS.5 + FS.6 + FS.nec) / CHE
Domestic Private Health Expenditure (PVT-D) Per Capita in US$	PVTD_PC_USD	(FS.4 + FS.5 + FS.6 + FS.nec) / Population / XRT
Domestic Private Health Expenditure (PVT-D) Per Capita in PPP Int$	PVTD_PC_PPP	(FS.4 + FS.5 + FS.6 + FS.nec) / Population / PPP
Voluntary Health Insurance (VHI) Prepayments as % Current Health Expenditure (CHE)	VHI%CHE	FS.5 / CHE
Out of Pocket Expenditure (OOP) as % Current Health Expenditure (CHE)	OOP%CHE	HF.3 / CHE
Out of Pocket Expenditure (OOP) as % Private Final Consumption (PC)	OOP%PFC	HF.3 / PFC
Out-of-Pocket Expenditure (OOP) Per Capita in US$	OOP_PC_USD	HF.3 / Population / XRT
Out-of-Pocket Expenditure (OOP) Per Capita in PPP Int$	OOP_PC_PPP	HF.3 / Population / PPP
Health Expenditure from External sources (EXT) as % of Current Health Expenditure (CHE)	EXT%CHE	(FS.2 + FS.7) / CHE
Health Expenditure from External Sources (EXT) per Capita in US$	EXT_PC_USD	(FS.2 + FS.7) / Population / XRT
Health Expenditure from External Sources (EXT) per Capita in PPP Int$	EXT_PC_PPP	(FS.2 + FS.7) / Population / PPP
Expenditure by FINANCING ARRANGEMENTS		
Compulsory Financing Arrangements (CFA) as % of Current Health Expenditure (CHE)	CFA%CHE	HF.1 / CHE
Government Financing Arrangements (GFA) as % of Current Health Expenditure (CHE)	GFA%CHE	HF1.1 / CHE
Compulsory Health Insurance (CHI) as % of Current Health Expenditure (CHE)	CHI%CHE	HF1.2 / CHE
Social Health Insurance (SHI) as % of Current Health Expenditure (CHE)	SHI%CHE	HF.1.2.1 / CHE
Compulsory Private Health Insurance (CHI-PVT) as % of Current Health Expenditure (CHE)	CHI_PVT%CHE	HF.1.2.2 / CHE
Voluntary Financing Arrangements (VFA) as % of Current Health Expenditure (CHE)	VFA%CHE	(HF.2 + HF.3) / CHE
Subsidy to insurance % Social health insurance schemes	TRAN%SHI	(HF.1.2.1 – FS.3) / HF.1.2.1
PHC INDICATORS		
Primary Health Care (PHC) Expenditure Per Capita in US$	PHC_PC_USD	PHC /Population / XRT
Primary Health Care (PHC) Expenditure as % Current Health Expenditure (CHE)	PHC%CHE	PHC / CHE
Domestic General Government Expenditure on Primary Health Care (PHC-G) as % Domestic General Government Health Expenditure (GGHED)	PHC_G %GGHED	PHC_GGHED (FS.1+FS.3) / GGHED
Domestic General Government Expenditure on Primary Health Care (PHC-G) as % Primary Health Care (PHC)	PHC_G%PHC	PHC_GGHED (FS.1+FS.3) / PHC
Externally Funded Primary Health Care Expenditure (PHC-EXT) as % Primary Health Care (PHC)	PHC_EXT%PHC	PHC_EXT (FS.2+ FS.7) / PHC
Primary Health Care (Government and Donors) as % Gross Domestic Product (GDP)	PHC_PUBLIC%GDP	(PHC_GGHED +PHC_EXT) / GDP
Disease INDICATORS		
Domestic General Government Expenditure (GGHED) on infectious and Parasitic diseases (DIS.1) as % GGHED	DIS.1_G%GGHED	DIS.1_GGHED / GGHED
Domestic General Government Expenditure (GGHED) on HIV/AIDS and Sexually Transmitted Diseases (DIS.1.1) as % GGHED	DIS.1.1_G %GGHED	DIS.1.1_GGHED / GGHED
Domestic General Government Expenditure (GGHED) on tuberculosis (DIS.1.2) as % GGHED	DIS.1.2_G %GGHED	DIS.1.2_GGHED / GGHED
Domestic General Government Expenditure (GGHED) on malaria (DIS.1.3) as % GGHED	DIS.1.3_G %GGHED	DIS.1.3_GGHED / GGHED
Domestic General Government Expenditure (GGHED) on reproductive health (DIS.2) as % GGHED	DIS.2_G %GGHED	DIS.2_GGHED / GGHED
Domestic General Government Expenditure (GGHED) on maternal conditions (DIS.2.1) as % GGHED	DIS.2.1_G %GGHED	DIS.2.1_GGHED / GGHED

Domestic General Government Expenditure (GGHED) on contraceptive management (family planning) (DIS.2.3) as % GGHED	DIS.2.3_G %GGHED	DIS.2.3_GGHED / GGHED
Domestic General Government Expenditure (GGHED) on nutritional deficiencies (DIS.3) as % GGHED	DIS.3_G%GGHED	DIS.3_GGHED / GGHED
Domestic General Government Expenditure (GGHED) on noncommunicable diseases (DIS.4) as % GGHED	DIS.4_G%GGHED	DIS.4_GGHED / GGHED
Domestic General Government Expenditure (GGHED) on injuries (DIS.5) as % GGHED	DIS.5_G%GGHED	DIS.5_GGHED / GGHED
Domestic General Government Expenditure (GGHED) on immunization programmes (HC.6.2) as % GGHED	HC.6.2_G%GGHED	HC.6.2_GGHED /GGHED
Health Expenditure from External sources (EXT) on infectious and parasitic diseases (DIS.1) as % EXT	DIS.1_EXT%EXT	DIS.1_EXT/ EXT
Health Expenditure from External sources (EXT) on HIV/AIDS and sexually transmitted diseases (DIS.1.1) as % EXT	DIS.1.1_EXT%EXT	DIS.1.1_EXT/EXT
Health Expenditure from External sources (EXT) on tuberculosis (DIS.1.2) as % EXT	DIS.1.2_EXT%EXT	DIS.1.2_EXT/ EXT
Health Expenditure from External sources (EXT) on malaria (DIS.1.3) as % EXT	DIS.1.3_EXT%EXT	DIS.1.3_EXT/ EXT
Health Expenditure from External sources (EXT) on reproductive health (DIS.2) as % EXT	DIS.2_EXT%EXT	DIS.2_EXT/ EXT
Health Expenditure from External sources (EXT) on maternal conditions (DIS.2.1) as % EXT	DIS.2.1_EXT%EXT	DIS.2.1_EXT/ EXT
Health Expenditure from External sources (EXT) on contraceptive management (family planning) (DIS.2.3) as % EXT	DIS.2.3_EXT%EXT	DIS.2.3_EXT/ EXT
Health Expenditure from External sources (EXT) on nutritional deficiencies (DIS.3) as % EXT	DIS.3_EXT%EXT	DIS.3_EXT/ EXT
Health Expenditure from External sources (EXT) on noncommunicable diseases (DIS.4) as % EXT	DIS.4_EXT%EXT	DIS.4_EXT/ EXT
Health Expenditure from External sources (EXT) on injuries (DIS.5) as % EXT	DIS.5_EXT%EXT	DIS.5_EXT/ EXT
Health Expenditure from External sources (EXT) on immunization programmes (HC.6.2) as % EXT	HC.6.2_EXT%EXT	HC.6.2_EXT/EXT

* Capital health expenditure only includes gross fixed capital investments in almost all countries.
